# Clinical Profile of Patients Admitted With Venous Thrombosis to a Tertiary Care Hospital in India

**DOI:** 10.7759/cureus.102603

**Published:** 2026-01-29

**Authors:** Bibin George, Sujeet Raina, Rajesh Sharma, Narvir S Chauhan, Krishna B Reddy

**Affiliations:** 1 Internal Medicine, All India Institute of Medical Sciences, Madurai, IND; 2 Internal Medicine, Dr. Rajendra Prasad Government Medical College, Tanda, IND; 3 Internal Medicine, EBM Day Care Clinic, Kangra, IND; 4 Radiology, All India Institute of Medical Sciences, Bilaspur, IND; 5 Internal Medicine, Apollo One, Apollo Hospitals, Chennai, IND

**Keywords:** cerebral venous thrombosis, d-dimer, deep vein thrombosis, mesenteric thrombosis, modified well's score, pulmonary embolism

## Abstract

Background and objectives: Venous thrombosis is a frequent cause of hospitalization worldwide; however, data describing its clinical profile in Indian patients, particularly from hilly regions, remain limited. The present study aimed to describe the clinical profile of patients admitted with venous thrombosis to the medical wards of a tertiary care hospital in Himachal Pradesh, India. Additionally, we examined the prevalence of elevated D-dimer levels across different venous thrombosis sites and assessed the proportion of patients with confirmed deep vein thrombosis (DVT) and pulmonary embolism who would have been retrospectively classified as “likely” using the modified Wells criteria.

Methods: This cross-sectional observational study was conducted over a one-year period. All adult patients admitted with clinically suspected and radiologically confirmed venous thrombosis who met the inclusion and exclusion criteria were included. Clinical characteristics were described, and the prevalence of elevated D-dimer levels across different venous thrombosis sites was assessed. The modified Wells pre-test probability scores were applied retrospectively to patients with confirmed DVT and pulmonary embolism to determine the proportion classified as “likely.”

Results: A total of 76 patients admitted with venous thrombosis were enrolled in the study. The mean age of the patients was 47.4 ± 16.3 years, and the female to male ratio was 1.3:1. Most patients had DVT (64.5%) (n=49) followed by pulmonary thromboembolism (PTE) (15.8%) (n=12) and cerebral venous thrombosis (CVT) (11.8%) (n=9) either as an isolated event or in combination with other venous thrombosis. Out of 49 DVT patients, the left lower limb was most commonly affected (75%) (n=36), and the most common presentation was limb swelling (95.9%) (n=47). The most common vessel involved was the femoropopliteal vein (38.8%) (n=19). Elevated D-dimer levels were observed in 91.8% (95% CI, 80.4-97.73%), 88.9% (95% CI, 51.75-99.72%), 100% (95% CI, 73.54-100%), and 81.82% (95% CI, 48.22-97.72%) of patients with DVT, CVT, PTE, and other thrombosis sites, respectively. When the modified Wells scores were applied retrospectively, 46 of 49 patients with DVT (93.9%; 95% CI: 83.13-98.72%) were classified as “Likely DVT” (score ≥2), while three patients (6.1%) were classified as “Unlikely DVT”. Among the 12 patients with PTE, 11 patients (91.7%; 95% CI: 61.52-99.79%) were classified as “Likely PTE” according to the modified Wells criteria.

Conclusion: Venous thrombosis was a common cause of admission, with isolated DVT being the most frequent presentation, followed by PTE and CVT. Smoking and malignancy were commonly observed in this cohort, reflecting their prevalence rather than independent risk associations. Most patients with confirmed DVT and PTE would have been retrospectively classified as “likely” by the modified Wells criteria; however, this represents case-based classification and not validation of the model. Further prospective studies are needed to evaluate diagnostic tools and risk factors.

## Introduction

Venous thrombosis is a common cause of hospitalization globally and venous thromboembolism (VTE) represents the third most common cause of death from cardiovascular disease worldwide, after myocardial infarction and stroke [1]. Thrombus can be defined as an aggregate of coagulated blood containing platelets, fibrin, and entrapped cellular elements within the vascular system, in accordance with standard pathology definitions. Deep vein thrombosis (DVT) is a blood clot that usually forms within the deep veins of the leg, but it can occur in the veins of the arms as well as the mesenteric, thoracic, cerebral, hepatic, renal, and portal veins. The disruption of the clot through the venous circulation can cause blockage in the pulmonary vasculature, resulting in fatal pulmonary embolism (PE). DVT leading to post-thrombotic or post-phlebitis syndrome, as long-term sequelae, can cause significant morbidity to the patient. Clinical symptoms and signs of venous thrombosis are non-specific. A high index of suspicion and validated clinical prediction rules like Wells criteria for PE help in categorizing patients with pre-test probability. VTE can be a consequence of acquired risk factors, referred to as a provoked event, or lack such provoking risk factors, referred to as an unprovoked event [2]. Most Indian data on venous thrombosis originate from urban or low-altitude settings, with limited representation from hilly regions such as Himachal Pradesh. Regional variations in geography, lifestyle, and healthcare access may influence risk factors and clinical presentation. This study aims to address this gap by characterizing the clinical profile of venous thrombosis involving multiple venous sites in patients from a tertiary center in this region. D-dimer levels were analyzed as the proportion of confirmed cases showing elevation across different venous thrombosis sites. The performance of the modified Wells pre-test probability score in classifying confirmed cases of DVT and PE as likely or unlikely was evaluated [3,4].

## Materials and methods

Study setting

This cross-sectional observational study was conducted in a tertiary care referral hospital in Kangra district, Himachal Pradesh, India.

Patient selection and data collection

All patients diagnosed with venous thrombosis over a one-year period (January 2019 to January 2020) fulfilling the inclusion and exclusion criteria were recruited using consecutive (total enumeration) sampling. All patients above the age of 18 years who were diagnosed with venous thrombosis of any site in medical wards and were willing to participate in this study were included. Exclusion criteria included patients younger than 18 years of age and those who declined to provide informed consent. Diagnosis was based on clinical presentation and radiological confirmation using standard imaging modalities: compression ultrasound or venous duplex sonography for upper and lower limb venous thrombosis, magnetic resonance venography for cerebral venous thrombosis (CVT), CT pulmonary angiography for pulmonary thromboembolism (PTE), and contrast-enhanced CT abdomen for abdominal or mesenteric venous thrombosis. DVT was defined by venous non-compressibility with or without intraluminal thrombus on ultrasonography; CVT by absent flow or filling defects in cerebral venous sinuses on MR venography; and PTE by intraluminal filling defects within the pulmonary arterial tree on CT pulmonary angiography. Data were collected using a structured proforma that recorded demographic variables, clinical presentation, comorbidities and predisposing risk factors, laboratory parameters including D-dimer levels, and thrombophilia evaluation where indicated, which included serum homocysteine levels, protein C and protein S activity, antithrombin III levels, antiphospholipid antibody testing, factor V Leiden mutation, and prothrombin gene mutation analysis, along with radiological findings, anatomical site of thrombosis, and final confirmed diagnosis. D-dimer levels were measured at admission using a quantitative immunoassay as part of routine laboratory evaluation. A value of ≥0.5 µg/mL was considered positive. Age-adjusted D-dimer cut-offs were not applied. D-dimer levels were analyzed descriptively as the proportion of patients with confirmed venous thrombosis who demonstrated elevated values across different thrombosis sites (DVT, PE, CVT, and other sites). Performance of pre-test probability score by modified Wells criteria in predicting cases of DVT and PE was assessed retrospectively, based solely on clinical variables documented at initial presentation and prior to radiological confirmation, to minimize information bias. A score of 2 or higher indicated that DVT was likely, while a score of less than 2 indicated that it was unlikely. A score of 4 or more indicated that PE was likely, while one of less than 4 indicated that it was unlikely [3,4].

Data analysis

Data were entered into Microsoft Excel (Redmond, WA, USA). Quantitative variables were expressed as means with standard deviations and categorical variables as frequencies and percentages. For D-dimer levels and Wells scores, results are presented as proportions with 95% confidence intervals (CIs), calculated using the Wilson method. No formal diagnostic accuracy measures requiring non-diseased controls were estimated.

## Results

During the study period, 76 patients admitted with venous thrombosis were enrolled in the study. Patients’ mean age was 47.4 ± 16.3 years, and they ranged in age from 24 to 92. Of the 76 patients, 44 (57.9%) were females, and 32 (42.1%) were males. The female to male ratio was 1.3:1. The mean age of males was 46.0 ± 15.2 years, while that of females was 48.5 ± 17.1 years. The distribution of age and thromboembolism types is depicted in Table 1.

**Table 1:** Distribution of thromboembolism based on age group (n=76) DVT, deep vein thrombosis; PTE, pulmonary thromboembolism; CVT, cerebral venous thrombosis; PVT, portal venous thrombosis; IVC, inferior vena cava; SVT, splenic venous thrombosis.

Age Group & Thrombus Site	21-30	31-40	41-50	51-60	61-70	>70	Total
DVT	3	13	8	7	8	5	44
PTE	1	1	4	2	0	1	9
CVT	3	2	2	0	0	0	7
DVT + PTE	1	0	1	0	0	1	3
Isolated PVT	1	1	1	0	0	0	3
DVT + CVT	0	0	1	0	1	0	2
Isolated SVT	0	1	0	1	0	0	2
PVT+SVT	0	0	1	0	1	0	2
Isolated IVC	0	1	0	0	0	0	1
PVT + IVC	0	0	0	1	0	0	1
PVT + SVT	0	1	0	0	0	0	1
Superior Mesenteric	1	0	0	0	0	0	1
Total	10	20	18	11	10	7	76

**Figure 1:**
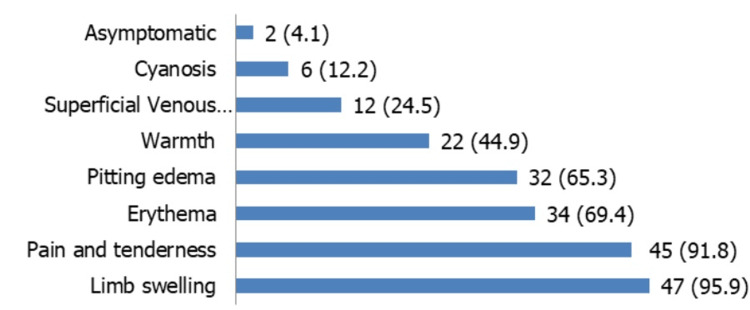
Clinical presentations of deep vein thrombosis (DVT) (n=49) *Values are expressed as frequency and percentage [n (%)]

Most patients had DVT (64.5%) (n=49), followed by PTE (15.8%) (n=12) and CVT (11.8%) (n=9) either as an isolated event or in combination with other venous thrombosis. Most males had DVT (46.9%) (n=15), followed by PTE (25%) (n=8), portal venous thrombosis (PVT) + splenic venous thrombosis (SVT) (6.3%) (n=2), and CVT (6.2%) (n=2). Among females, DVT was most common (65.9%) (n=29), followed by CVT (20.6%) (n=7) and isolated PVT (6.8%) (n=3). Out of 49 patients with DVT, 48 patients had lower limb DVT (97.5%), and one had upper limb DVT (2.1%). The left lower limb was most commonly affected (75%) (n=36). Out of the 49 patients, 40 had provoked DVT (81.6%), and nine had no provoking factor (18.4%). The distribution of clinical symptoms and signs in patients with DVT is shown in Figure 1.

The distribution of vessels involved in DVT is depicted in Table 2.

**Table 2:** Distribution based on vessels involved (n=49) GSV, great saphenous vein.

Vessels involved	Frequency	Percentage
Femoral + popliteal vein	19	38.8
Isolated femoral vein	11	22.4
Isolated popliteal vein	9	18.4
Infrapopliteal vein	2	4.1
Femoral + GSV	2	4.1
Popliteal + infrapopliteal vein	2	4.1
Common iliac + external iliac + femoral	1	2.0
External iliac + femoral + popliteal+ GSV	1	2.0
Infrapopliteal + short saphenous vein	1	2.0
Axillary vein	1	2.0
Anterior tibial vein	0	0.0
Posterior tibial vein, peroneal vein	0	0.0

Of the 49 patients, 45 had deep sites (91.8%) and four had superficial + deep sites (8.2%). Moreover, 44 patients had proximal (89.8%), three had distal (6.1%), and two had proximal + distal (4.1%) DVT sites.

In CVT patients, the transverse sinus (44.4%) (n=4) was most involved, followed by the superior sagittal sinus (33.3%) (n=3), while four patients (44.4%) had multiple sinuses involved. The left side of the brain was involved in 66.7% (n=6), while the rest had right side involvement (33.3%) (n=3). Four of the patients (44.4%) had nonhemorrhagic infarcts. The clinical presentation of patients with CVT is shown in Table 3.

**Table 3:** Clinical presentations of cerebral venous thrombosis (CVT) (n=9)

Presentation	Frequency	Percentage
Headache	5	55.6
Vomiting	3	33.3
Focal deficits	2	22.2
Convulsions	1	11.1
Altered sensorium	1	11.1
Fever	1	11.1

Among the 12 PTE patients, the most common clinical presentation was dyspnea (91.7%) (n=11), followed by tachycardia (91.7%) (n=11). The most common ECG finding in PTE patients was sinus tachycardia alone (41.7%) (n=5), followed by sinus tachycardia + S1Q3T3 pattern (25%) (n=3). The distribution of vessels involved in PTE is shown in Table 4.

**Table 4:** Distribution based on vessels involved in pulmonary thromboembolism (PTE) (n=12)

Vessels involved in PTE	Frequency	Percentage
Isolated main pulmonary artery	7	58.3
Isolated segmental branches	2	16.7
Isolated sub-segmental branches	1	8.3
Segmental + lobar artery	1	8.3
Pulmonary + segmental + lobar artery	1	8.3
Isolated lobar pulmonary artery	0	0.0

Common predisposing factors for venous thrombosis are presented in Table 5.

**Table 5:** Distribution based on predisposing factors DVT, deep vein thrombosis; PTE, pulmonary thromboembolism; CVT, cerebral venous thrombosis; OCP, oral contraceptive pills; RHD, rheumatic heart disease; CAD, coronary artery disease; HAP, hospital acquired pnuemonia; CAP, community acquired pnuemonia; LSCS, lower segment cesarian section; COPD, chronic obstructive pulmonary disease; DCMP, dilated cardiomyopathy; HIV, human immunodeficiency virus; HCV, hepatitis C virus; SLE, systemic lupus erythematosus.

Predisposing Factors	DVT [n=49] n (%)	PTE [n=12] n (%)	CVT [n=9] n (%)	Other [n=11] n (%)	Total [n=76] n (%)
Smokers	13 (26.5)	5(41.7)	1 (11.1)	6 (66.7)	25(32.9)
Tobacco chewers	3 (6.1)	0	0	0	3 (3.9)
Steroid abuse	1 (2)	0	0	0	1 (1.3)
OCP use	1 (2)	0	0	0	1 (1.3)
Hypertension	7 (14.3)	3 (25)	0	2 (22.2)	12(15.8)
Hyperlipidemia	7 (14.3)	2(16.7)	1 (11.1)	0	10(13.2)
Diabetes	8 (16.3)	2(16.7)	0	2 (22.2)	12(15.8)
RHD	2 (4.1)	0	0	0	2 (2.6)
CAD	1 (2)	0	0	0	1 (1.3)
Heart failure	2 (4.1)	0	0	0	2 (2.6)
Atherosclerotic peripheral artery disease	2 (4.1)	0	0	0	2 (2.6)
Stroke or transient ischemic attack	2 (4.1)	0	1 (11.1)	0	3 (3.9)
Chronic kidney disease	3 (6.1)	1 (8.3)	0	0	4 (5.3)
Gastrointestinal bleed	0	0	0	4 (44.4)	4 (5.3)
Sepsis	6 (12.2)	4(33.3)	0	0	10(13.2)
a. Pneumonia (HAP/CAP)	2	4			
b. Klebsiella	1	1			
c. Klebsiella + Pseudomonas	1	3			
d. Urosepsis	2	0			
e. Rickettsial	2	0			
Varicose veins	2 (4.1)	0	0	0	2 (2.6)
Pregnancy	0	0	0	0	0
LSCS	4 (8.2)	1 (8.3)	0	0	5 (6.6)
Chronic liver disease	0	0	0	2 (22.2)	2 (2.6)
COPD	1 (2)	2(16.7)	0	0	3 (3.9)
DCMP	2 (4.1)	0	0	0	2 (2.6)
Hypothyroidism	3 (6.1)	0	0	0	3 (3.9)
Hematological disorder	2 (8.2)	0	2 (22.2)	0	4 (5.3)
a. Acute leukemia	1	0	1		
b. Multiple myeloma	1	0	1		
Malignancy	8 (16.3)	2(16.7)	4 (44.4)	2 (22.2)	16(21.1)
Recent surgery	4 (8.2)	0	0	0	4 (5.3)
Recent immobilization	3 (6.1)	1 (8.3)	0	0	4 (5.3)
Fracture shaft of femur	1 (2)	0	0	0	1 (1.3)
Previous DVT prophylaxis	3 (6.1)	1 (8.3)	0	0	4 (5.3)
HIV infection	1 (2)	0	0	0	1 (1.3)
HCV infection	0	0	0	1 (11.1)	1 (1.3)
Tuberculosis	4 (8.2)	4(33.3)	0	1 (11.1)	9 (11.8)
Pulmonary	4	4		0	
Extrapulmonary	0	0		1	
Obesity	10(20.4)	3 (25)	1 (11.1)	2 (22.2)	16(21.1)
Overweight	8 (16.3)	0	0	2 (22.2)	10(13.2)
Connective tissue disorder	4 (8.2)	0	0	0	4 (5.3)
a. SLE	2				
b. Systemic sclerosis	1				
c. Mixed connective tissue disorder	1				
Thrombophilia profile				0	
a. Hyperhomocysteinemia	6 (12.2)	0	3 (33.3)		9(11.8)
b. Protein C deficiency	0	0	0		0
c. Protein S deficiency	1 (2)	0	0		1(1.3)
d. Prothrombin gene mutation	0	0	0		0
e. Factor V laden mutation	0	0	0		0
f. Antiphospholipid antibody syndrome	2	0	0		2(2.6)
g. Hypofibrinogenemia	0	1 (8.3)	1(11.1)		2(2.6)

Elevated D-dimer levels were observed in 91.8% (95% CI, 80.4-97.73%), 88.9% (95% CI, 51.75-99.72%), 100% (95% CI, 73.54-100%), and 81.82% (95% CI, 48.22-97.72%) of patients with DVT, CVT, PTE, and other thrombosis sites, respectively, as depicted in Table 6.

**Table 6:** Proportion of confirmed venous thrombosis cases with elevated D-dimer levels

Venous thromboembolism type	Elevated D-dimer (n)	Total cases (N)	Proportion (%)
Deep vein thrombosis	45	49	91.8
Pulmonary thromboembolism	12	12	100
Cerebral venous thrombosis	8	9	88.9
Other venous thrombosis	9	11	81.8

When the modified Wells scores were applied retrospectively, 46 of 49 patients with DVT (93.9%; 95% CI: 83.13-98.72%) were classified as “Likely DVT” (score ≥2), while three patients (6.1%) were classified as “Unlikely DVT”. Among the 12 patients with PTE, 11 patients (91.7%; 95% CI: 61.52-99.79%) were classified as “Likely PTE” according to the modified Wells criteria.

## Discussion

VTE, comprising DVT and PE, can result in significant mortality, morbidity, and healthcare expenditure. Approximately one-third of patients with symptomatic VTE manifest as PE, whereas two-thirds manifest as DVT alone. Both DVT and PE can be clinically silent (asymptomatic) and hence may not be suspected [5]. In our study, the mean age of patients with venous thrombosis was 47.4±16.3 years; 60.5% (n=46) were aged above 40 years, 26% (n=20) were aged between 31 and 40 years, and 14% (n=10) were aged between 21 and 30 years. These data align with those of other studies [6-8]. Females predominated over males with a ratio of 1.3:1, which is in concordance with studies by Naqvi et al. and Garg et al. but differs from some Indian subgroup studies [8-10]. In our study, DVT (72.7%) (n=32/44) and CVT (77.8%) (n=7/9) were most common among females, while PTE was most common among males (75%) (n=9/12). Our findings are in concordance with Agarwal et al. and Garg et al. [10,11]. The female predominance observed in our study can be attributed to pregnancy and the postpartum period, including cases following caesarean section, a higher proportion of CVT among women, and the presence of hyperhomocysteinemia likely related to nutritional deficiencies. As this was a hospital-based study, referral patterns may also have influenced the gender distribution.

The lower limb was most commonly involved (n=48/49) (97.9%), and one patient had upper limb DVT (n=1/49) (2.1%). Among lower limb DVTs, the left lower limb (75%) (n=36) was the most common. These findings are in line with those of other Indian studies [12,13]. Virchow suggested that this disparity may be related to compression of the left common iliac vein by the right common iliac artery [14]. We observed that proximal DVT was more common, occurring in 89.7% (n=44) of patients, whereas isolated distal DVT was seen in 6.1% (n=3); an additional 4.1% (n=2) of patients had involvement of both proximal and distal veins. Limb swelling was the most common symptom (95.7%) (n=47), and the femoropopliteal vein was most commonly involved (38.8%) (n=19). These results are consistent with other Indian and Western studies [8,15-18].

Among the 76 patients with venous thrombosis, the most common predisposing factors found were as follows: smoking (33%) (n=25), followed by obesity and malignancy (21% (n=16) each), diabetes and hypertension (16% (n=13) each), hyperlipidemia and sepsis (13% (n=10) each), tuberculosis and hyperhomocysteinemia (11.8% (n=9) each), postpartum period (6.6% (n=5)), and hematological disorder, recent surgery and immobilization, connective tissue disorders, and chronic kidney disease (CKD) (5.3% (n=4) each). We observed that 26.5% (n=13/49) of DVT patients, 41.7% (n=5/12) of PTE patients, 11.1% (n=1/9) of CVT patients, and 66.7% (n=6/11) of other thromboembolism site patients were smokers. Several prospective studies have reported smoking to be an independent risk factor [19,20], whereas others have failed to detect a significant relationship between smoking and VTE. As this was a descriptive study without a control group, these findings reflect the prevalence of smoking within the cohort and do not permit inference regarding smoking as an independent risk factor for VTE. We observed that 10 patients (13.2%) with venous thrombosis had underlying sepsis. Among patients with sepsis, six had pneumonia, two had urosepsis, and two had rickettsial infections. The pneumonic illnesses included both community-acquired and hospital-acquired pneumonia. In these patients, venous thrombosis occurred in the clinical context of acute infection and hospitalization, often accompanied by factors such as immobilization, systemic inflammation, hypoxia, and dehydration. Coexisting factors such as smoking were also commonly observed, as reported in previous observational studies [21]. Given the descriptive nature of the present study, these findings do not establish independent causality. Rickettsial infection may cause a hypercoagulable state, leading to disseminated intravascular coagulation and DVT in severe infection cases during the acute phase [22]. Regarding thrombophilia profile, nine patients (11.84%) with hyperhomocysteinemia (six DVT and three CVT), two with antiphospholipid antibody syndrome (2.6%), and one with protein C deficiency were found. These data are in contrast with other studies, and the higher rate of hyperhomocysteinemia might be due to the concomitant vitamin B12 deficiency observed in our population [23].

As in other studies, none of the patients with chronic liver disease had DVT, CVT, or PTE, while chronic liver disease contributed to 22% (n=2) of mesenteric thrombosis cases. This can be attributed to the protective antithrombotic mechanisms in liver disease [24]. All five patients who had developed venous thrombosis during the postpartum period were in the first month after delivery, and all had undergone lower segment caesarean section (LSCS). It is suggested that puerperal CVT is 10-12 times more frequent in India and the predisposing factors, like anemia, increased coagulability of blood, slowing of the blood stream, and dehydration, aggravated in puerperium, contributed to the high incidence of CVT. Caesarean sections further increase the risk due to the postsurgical decline of protein C levels caused by surgically induced tissue damage. This causes activation of blood clotting with increased thrombin generation, which in turn activates protein C and promotes its clearance from the plasma [25]. Out of 16 malignancy patients (21%), 50% (n=8) had DVT, 25% (n=4) had CVT, and 12.5% (n=2) each had PTE and other VTE types.

The proportion of patients with elevated D-dimer levels among those diagnosed with DVT, PTE, and CVT, as well as the distribution of patients classified as “likely DVT” and “likely PTE” using the modified Wells criteria, was comparable to that reported in previous studies [26-28]. In this case-series, retrospective application of the modified Wells model demonstrated that most patients with confirmed DVT or PTE fell into the “likely” category; however, these findings are descriptive and do not constitute validation of diagnostic performance. Among the seven patients with D-dimer levels <0.5 µg/mL, three had DVT, one had CVT, and two had PVT. VTE can occur with a negative D-dimer concentration and related to methodological limitations. However, the lack of increase in D-dimer levels could also be caused by fibrinolysis alteration. This can also be attributed to the thrombus volume and chronicity [29].

Limitations

This was a single-center, hospital-based study, which may limit the generalizability of the findings. The sample size was modest, especially for subgroups such as CVT, PTE, and other uncommon thrombosis sites, reducing the power of subgroup analyses. The cross-sectional design precluded assessment of outcomes such as recurrence, long-term complications, and mortality. The absence of a control or non-VTE comparison group is the most significant limitation, as it precludes assessment of independent risk factors. As the study included only patients with radiologically confirmed VTE, formal diagnostic accuracy measures (specificity, false-positive rates, and predictive values) for D-dimer and Wells scores could not be assessed. The findings therefore represent the proportion of confirmed cases with elevated D-dimer levels or classified as “likely” by Wells criteria, rather than true diagnostic sensitivity in a suspected population. Well's scores were applied retrospectively, which may have introduced information bias. Thrombophilia testing was not performed uniformly in all patients; it was selectively done when no clear provoking factor was identified in order to avoid unnecessary financial burden, which may have led to underestimation of inherited or acquired prothrombotic states.

## Conclusions

This study describes the clinical profile of patients admitted with venous thrombosis in the medical wards of a tertiary care hospital. Venous thrombosis was not an uncommon presentation, with most patients having isolated DVT, followed by PTE and CVT. The cohort predominantly comprised younger patients with a female preponderance. Most of the patients had isolated DVT followed by PTE and CVT. Smoking and malignancy were commonly observed among patients with venous thrombosis in this cohort. Elevated D-dimer levels and classification as “likely” by the modified Wells criteria were frequent among patients with confirmed disease, reflecting case-based positivity rather than diagnostic accuracy or validation of these tools.

Early diagnosis and treatment of VTE can essentially reduce morbidity and mortality. More prospective studies are required on this topic to draw more evidence and add to the existing data.
